# Methylome and Transcriptome-Based Integration Analysis Identified Molecular Signatures Associated With Meningitis Induced by *Glaesserella parasuis*

**DOI:** 10.3389/fimmu.2022.840399

**Published:** 2022-02-25

**Authors:** Ling Guo, Hongxing Cheng, Shulin Fu, Jun Liu, Yunfei Zhang, Yinsheng Qiu, Hongbo Chen

**Affiliations:** ^1^ Hubei Key Laboratory of Animal Nutrition and Feed Science, Wuhan Polytechnic University, Wuhan, China; ^2^ Hubei Collaborative Innovation Center for Animal Nutrition and Feed Safety, Wuhan Polytechnic University, Wuhan, China

**Keywords:** *Glaesserella parasuis*, meningitis, inflammation, Whole-genome DNA methylation, transcriptome

## Abstract

*Glaesserella parasuis* (*G. parasuis*) can elicit serious inflammatory responses and cause meningitis in piglets. Previous epigenetic studies have indicated that alterations in host DNA methylation may modify the inflammatory response to bacterial infection. However, to date, genome-wide analysis of the DNA methylome during meningitis caused by *G. parasuis* infection is still lacking. In this study, we employed an unbiased approach using deep sequencing to profile the DNA methylome and transcriptome from *G. parasuis* infected porcine brain (cerebrum) and integrated the data to identify key differential methylation regions/sites involved in the regulation of the inflammatory response. Results showed that DNA methylation patterns and gene expression profiles from porcine brain were changed after *G. parasuis* infection. The majority of the altered DNA methylation regions were found in the intergenic regions and introns and not associated with CpG islands, with only a low percentage occurring at promoter or exon regions. Integrated analysis of the DNA methylome and transcriptome identified a number of inversely and positively correlated genes between DNA methylation and gene expression, following the criteria of |log_2_FC| > 0.5, |diffMethy| > 0.1, and *P* < 0.05. Differential expression and methylation of two significant genes, semaphoring 4D (*SEMA4D*) and von Willebrand factor A domain containing 1 (*VWA1*), were validated by qRT-PCR and bisulfite sequencing. Gene Ontology (GO) and Kyoto Encyclopedia of Genes and Genomes (KEGG) enrichment analyses demonstrated that DNA methylation inversely correlated genes in *G. parasuis* infected porcine brains were mainly involved with cell adhesion molecules (CAMs), bacterial invasion of epithelial cells, RIG-1-like receptor signaling pathways, and hematopoietic cell lineage signaling pathways. In addition, a protein-protein interaction network of differentially methylated genes found potential candidate molecular interactions relevant to the pathology of *G. parasuis* infection. To the best of our knowledge, this is the first attempt to integrate the DNA methylome and transcriptome data from *G. parasuis* infected porcine brains. Our findings will help understanding the contribution of genome-wide DNA methylation to the pathogenesis of meningitis in pigs and developing epigenetic biomarkers and therapeutic targets for the treatment of *G. parasuis* induced meningitis.

## Introduction

*Glaesserella parasuis* (*G. parasuis*), is a commensal pathogen found in the porcine upper respiratory tract, and can invade the bloodstream and cause polyserositis, arthritis, meningitis, and frequently, pneumonia like symptoms, and is the etiological agent of Glässer’s disease (1). To date, 15 serovars have been identified on the basis of immunodiffusion, using heat-stable antigen extracts, and numerous serovars do not induce protective cross-immunity (2). Epidemiological studies have shown that serovar 5 is the most prevalent form associated with this disease in China, followed by serovars 4, 7, 13, and 14 (3). Because of the high morbidity and mortality caused by *G. parasuis* infection, it has become a major threat to the swine industry throughout the world, and its control and prevention are urgently needed (4).

Meningitis, a common symptom of *G. parasuis* infection, is defined as inflammation of the meninges, and an abnormal number of white blood cells in the cerebrospinal fluid with specific clinical symptoms (5). Aseptic and bacterial meningitis vary significantly (6, 7), with most cases of aseptic meningitis being associated with viral infection and generally self-limited with a good prognosis. However, bacterial meningitis requires prompt diagnosis and medical treatment but even then mortality still occurs regardless of its acute or chronic onset (8, 9). *Streptococcus pneumoniae*, *Haemophilus influenzae* and *Neisseria* are the main bacterial pathogens responsible for acute bacterial meningitis (9, 10) and it is reported that the host genetic factors are the major determinants of susceptibility for bacterial meningitis (7). Recently, zoonotic meningitis has been occasionally linked to pig farmers who have developed acute bacterial meningitis after handling raw swine meat (11). At present, there remains an incomplete understanding of the pathogenesis and the best methods for the prevention and treatment of bacterial meningitis and further research looking at the pathogenesis of bacterial meningitis is urgently needed. Thus, research on the pathology of bacterial meningitis caused by *G. parasuis* using an infected porcine model appears to be meaningful for both the porcine industry and human public health.

DNA methylation is an epigenetic modification of DNA involving the addition of a methyl group onto the 5-carbon of cytosine to form 5-mehylcytosine (5mC), and represents a major regulator of gene expression, depending on the genomic context such as islands, transcriptional start sites, gene bodies, regulatory elements and repeat sequences (12, 13). Genome-wide DNA methylation patterns have been shown to be associated with gene transcription, cancer, mammalian embryo development, and a variety of diseases, including autoimmune and inflammatory disorders (14, 15). In a recent study of multiple sclerosis, and autoimmune disease, DNA methylation was reported to be involved in the process blood-brain barrier breakdown, inflammatory response, failure of myelination and neurodegeneration (16). This epigenetic modification has also been implicated in the pathogenesis of chronic inflammatory responses by regulating differentiation, proliferation, apoptosis, and activation of immune cells (17). Furthermore, the host DNA methylation profile can be altered upon exposure of cells to environmental pathogens, and these changes induced by the action of viral and bacterial infection may suppress the production of anti-inflammatory factors and activate the pro-inflammatory cells, as well as modify the immune response to bacterial infection (17, 18). Although lots of research has focused on the pathogenic mechanism of *G. parasuis*, little is known about alterations in host DNA methylation or the corresponding effects on the regulation of the transcriptome in meningitis under pathogenic conditions.

In the present study, we aimed to identify key differentially methylated regions/sites that may potentially regulate the function of gene expression during the process of *G. parasuis* infection, and these data may provide useful information for the development of epigenetic biomarkers and/or therapeutic targets for the treatment of *G. parasuis* induced meningitis. Thus, we examined the DNA methylation profile at a single base-pair resolution using whole-genome bisulfite sequencing and integrated the methylome information with transcriptome data from RNA-seq in normal and *G. parasuis* infected porcine brains.

## Materials and Methods

### Bacterial Strain and Growth Conditions

A highly virulent strain of serovar 5 (*G. parasuis* SH0165 strain), was isolated from the lungs of a commercial pig showing signs of arthritis, fibrinous polyserositis, hemorrhagic pneumonia, and meningitis (19). The SH0165 strain was cultured in tryptic soy broth (Difco Laboratories, Franklin Lakes, NJ, USA) or tryptic soy agar (Difco Laboratories) at 37°C, supplemented with 10 µg/mL of nicotinamide adenine dinucleotide (NAD) (Sigma-Aldrich Corporation, St. Louis, MO, USA) and 10% fetal bovine serum (Gibco, Carlsbad, CA, USA).

### Animals and Experimental Design

This study was conducted in strict accordance with the recommendations of the Chinese Regulations for the Administration of Affairs Concerning Experimental Animals 1988 and the Hubei Regulations for the Administration of Affairs Concerning Experimental Animals 2005. The protocol was ratified by the Hubei Province Science and Technology Department of China (permit number SYXK[ER] 2010-0029). Animal studies were approved by the Animal Care and Use Committee of Wuhan Polytechnical University, Hubei Province, China (EM947, 5^th^ November 2020). All experimental animals were euthanized at the end of the experiment.

Six 28-day-old naturally farrowed early weaned piglets (Duroc × Landrace × Large white), weighing 8-10 kg, were obtained from Wuhan Wannianqing Animal Husbandry Co., Ltd (Wuhan, China) for *in vivo* experimentation. All experimental piglets were negative for antibodies against *G. parasuis* when tested using INgezim Haemophilus 11.HPS.K.1 (INgezim, Spain).

The six piglets were randomly divided into two groups including the negative control group and infected group. The infected group was challenged intraperitoneally with 2 × 10^9^ CFU of the *G. parasuis* SH0165 strain in 1 mL of normal saline. The negative control group was injected intraperitoneally with an equivalent volume of saline alone. All the piglets were monitored for 7 days after challenge, and the cerebrum tissues from the infected and control groups were collected for whole-genome bisulfite sequencing and RNA-seq.

### DNA Preparation and Whole-Genome Bisulfite Sequencing

Genomic DNA from porcine brain tissue was extracted with Allprep DNA/RNA/Protein Mini Kit (Qiagen, Hilden, Germany) according to the manufacturer’s protocol. Then extracted DNA (1 µg) was spiked with 1 ng of fragmented unmethylated phage λ DNA (Promega, Madison, WI, USA, average size: 300 bp), which was end-repaired, A tailed, and adapter-ligated using Truseq DNA sample prep kit (Illumina Inc., USA) according to the manufacturer’s protocol. Adapter-ligated DNA was gel isolated and recovered using a QIAquick gel extraction kit (Qiagen, Hilden, Germany). After a cleanup with AMPure XP beads (Beckman Coulter Inc., Fullerton, CA, USA), bisulfite conversion was performed using EZ-DNA Methylation-Gold Kit (Zymo Research, Orange, CA, USA) according to the manufacturer’s protocol. Then, the bisulfite converted DNA was amplified with PfuTurbo Cx Hotstart DNA Polymerase (Agilent Technologies, Inc., Santa Clara, CA, USA) using the following thermal cycles: 95°C 5 min, 98°C 30 s, 12 cycles of (98°C 10 s, 65°C 30 s, 72°C 30 s), 72°C 5 min. Libraries were cleaned with AMpure XP beads and fragment length determined using an Agilent 2100 (Agilent Technologies, Inc., Santa Clara, CA, USA). Quantified libraries (concentration > 2 nM) were then sequenced using the Illumina HiSeq2500 platform.

### RNA Sequencing

Total RNA was extracted using the miRNeasy Mini Kit (Qiagen, Hilden, Germany) in accordance with the manufacturer’s instructions and RIN number assess for RNA integrity using an Agilent Bioanalyzer 2100 (Agilent Technologies, Inc., Santa Clara, CA, USA). Quantified total RNA was further purified using an RNAClean XP Kit (Beckman Coulter Inc., Fullerton, CA, USA) and RNase-Free DNase (Qiagen, Hilden, Germany). Only RNA with a RIN of ≥ 7.0 and an 28S/18S ratio of ≥ 0.7 were selected for deep sequencing. Libraries were generated using the VAHTS Total RNA-seq Library PrepKit for Illumina (Vazyme Biotech Co., Ltd., Nanjing, China) and were subsequently sequenced using the Illumina HiSeq X-Ten platform.

### Whole-Genome Bisulfite Sequencing Data Analysis

General quality control was performed using FastQC v 0.11.5 (http://www.bioinformatics.babraham.ac.uk/projects/fastqc/). Low quality reads (error ratio > 0.01 or read length < 70 bp or unpaired-reads) and adaptors were removed using Trimgalore v0.4.1 (http://www.bioinformatics.babraham.ac.uk/projects/trim_galore/) and Cutadapt v1.9.1 (20). Filtered datasets were aligned to the reference genome, Sus-Scrofa.Sscrofa 10.2, using Bismark v 0.15.0 (21), and using Bowtie2 v 2.2.6 (22) as the underlying alignment tool. Mapping for all datasets generated from the same library were merged, and duplicates removed *via* the Bismark deduplication tool. Mapped reads were then separated by genome (Sscrofa 10.2 or phage λ) and by source strand (plus or minus). The first four and last one base of each read2 in all read pairs was clipped due to positional methylation bias, and any redundant mapped bases due to overlapping mates from the same read pair were trimmed to avoid bias in the quantification of methylation status. Read pairs mapped to phage λ were used as a QC assessment to confirm that the observed bisulfite conversion rate was >99%. Read pairs mapped to the Sscrofa 10.2 reference genome were used for downstream analysis.

The R package DSS (Dispersion shinkage for sequencing data, DSS) was used to identify DML (Differential methylation loci, DML) with the callDML function, and DMR (Differential methylation region, DMR) with the callDMR function. DMRs that contain at least three validated CpG sites and a minimum length of 50 bp were retained for downstream analysis. DMLs and DMRs with a threshold *p* value < 0.05 and mean methylation difference > 0.1 were considered to be significantly differentially methylated.

The genomic context of the identified DMLs and DMRs with respect to known genes and gene features was analyzed by using the R package goldmine (http://jeffbhasin.github.io/goldmine). The function getCpgFeatures, getFeatures, and getGenes were used to obtain CpG islands, ENCODE supertracks, and RefSeq gene models from the database of the UCSC Genome Browser (23, 24).

### Transcriptome Data Analysis

The raw reads were filtered by using the Seqtk sequence processing tool (https://github.com/lh3/seqtk) and mapped to the reference genome (Sus-scrofa.Sscrofa10.2.dna.toplevel.fa) by using the Hisat2 alignment program (25) and StringTie was used to count the gene fragments (26, 27). Package edgeR (28) was used to identify differentially expressed genes with a threshold fold change > 2 and adjusted *p*-value < 0.05, which was considered as significantly differentially expressed.

### Integration of DNA Methylation and Gene Expression

To reveal the correlation between DNA methylation status and gene expression, we integrated DNA methylation and gene expression data. First, genes that were significantly differentially methylated in the promoter regions were selected with a threshold of mean methylation difference > 0.1 and *p* value < 0.05. Second, genes that were significantly differentially expressed were selected with a threshold of |log_2_FC| >0.5 and *p* value < 0.05. For these two selected gene sets, they were firstly merged by the merge function in R. Then, the correlation for the methylation difference and expression difference were determined by “cor. test” in R using Pearson method. Significantly negative correlations were considered if the correlation coefficient was negative and the *p* value was < 0.05, and significantly positive correlations were considered if the correlation coefficient was positive, and the *p* value was < 0.05. Differentially expressed genes that were negatively correlated with methylation were subjected to further studies.

### Gene Ontology (GO) and Kyoto Encyclopedia of Genes and Genomes (KEGG) Enrichment Analysis

To discover the potential biological function underlying the identified genes, we conducted GO and KEGG enrichment analysis on the differentially expressed or differentially methylated genes. Genes were mapped to each term within the GO database. GO terms with adjusted *p* values < 0.05 were considered as significantly enriched. Pathway annotation of the entire genome was conducted by the KEGG automatic annotation server. Pathways with *p* values ≤ 0.05 were deemed to be significantly enriched.

### PPI Network Construction

STRING database (ELIXIR, Hinxton, Cambridge shire, UK) were utilized for the protein-protein interaction (PPI) network construction, based on the methylation and expression of inversely correlated genes. Network Analyzer (http://www.mpi-inf.mpg.de/) was used to determine the topological properties of the biological networks. Cytoscape software (Institute for Systems Biology, Seattle, WA, USA) was used for network visualization.

### Bisulfite Sequencing

Bisulfite sequencing was performed to validate the DMRs detected by the whole-genome DNA methylation analysis. DNA (330 ng) was isolated using Quick-DNA Miniprep Kit (Zymo Research, Orange, CA, USA) and DNA bisulfite conversion was conducted by using the EZ DNA Methylation Gold Kit (Zymo Research, Orange, CA, USA). Bisulfite-converted DNA (1 µg) was amplified by PCR with EpiTaq polymerase (for bisulfite-treated DNA) (TaKaRa Biotechnology Co., Ltd., Dalian, China), and primers (**Supplementary File 1**) were manually designed using Primer Premier 5. Before primer design, DNA sequences need to be methylation converted in the MethPrimer website (http://www.urogene.org/methprimer/). PCR products were cloned using the Topo TA cloning kit (Invitrogen Corporation, Carlsbad, CA, USA) and after verification of successful cloning, at least ten different clones for each sample were sent for sequencing at TSINGKE Biological Technology. The DNA methylation profile of individual CpG sites were analyzed and presented using BiQ Analyzer (https://biq-analyzer.bioinf.mpi-inf.mpg.de/).

### Quantitative Real-Time RT-PCR

Total RNA was extracted from porcine brain tissues using RNeasy Mini Kit (Qiagen, Hilden, Germany), and reverse transcribed into cDNA using reverse transcriptase (TaKaRa Biotechnology Co., Ltd., Dalian, China). cDNA was further quantified using the SYBR Green PCR Kit (TaKaRa Biotechnology Co., Ltd., Dalian, China) according to the manufacturer’s protocol. Individual samples underwent three technical repeats, with β-actin as the reference gene. The Primer Premier 5.0 software was utilized to design the PCR primers (**Supplementary File 2**).

### Statistical Analysis

Experimental data were analyzed using the two-tailed Student’s *t*-test and presented as the mean ± standard deviation and differences with *P* < 0.05 were considered to be significant (^*^ *P* < 0.05; ^**^ *P* < 0.01).

## Results

### Comparative Analysis of Global DNA Methylation Patterns

Whole-genome bisulfite sequencing was performed with the Illumina HiSeq 2500 to explore the global DNA methylation changes associated with the pathology of meningitis induced by *G. parasuis*. A total of 626.50 ± 47.40 million and 627.76 ± 36.65 million raw reads were obtained from the infected and negative control groups, respectively. After filtration and quality control, the clean reads ratio ranged from 96.1% to 99.0% (**Supplementary File 3**). All the multi-mapped reads duplicated reads and adaptors were removed and aligned to the Sus*scrofa* genome, with the unique mapping ratio ranging from 54.3% to 60.7% (**Supplementary File 3**). Unmethylated phage λ DNA was added to evaluate the bisulfite conversion ratio, which ranged from 99.1% to 99.4% (**Supplementary File 3**).

Cytosine methylation levels at a single base-pair resolution were explored using callDML function in the R package DSS. Results showed that 316,621 CpG sites were differentially methylated in *G. parasuis* infected porcine brain, with 132,557 CpG sites hypermethylated and 184,064 CpG sites hypomethylated (*P* < 0.05) (**Supplementary File 4**). We also identified 12,145 (DMRs) that were statistically significant between the two groups, with 4,649 DMRs hypermethylated and 7,496 DMRs hypomethylated in *G. parasuis* infected porcine brain (*P* < 0.05) (**Supplementary File 5**).

**Figure 1:**
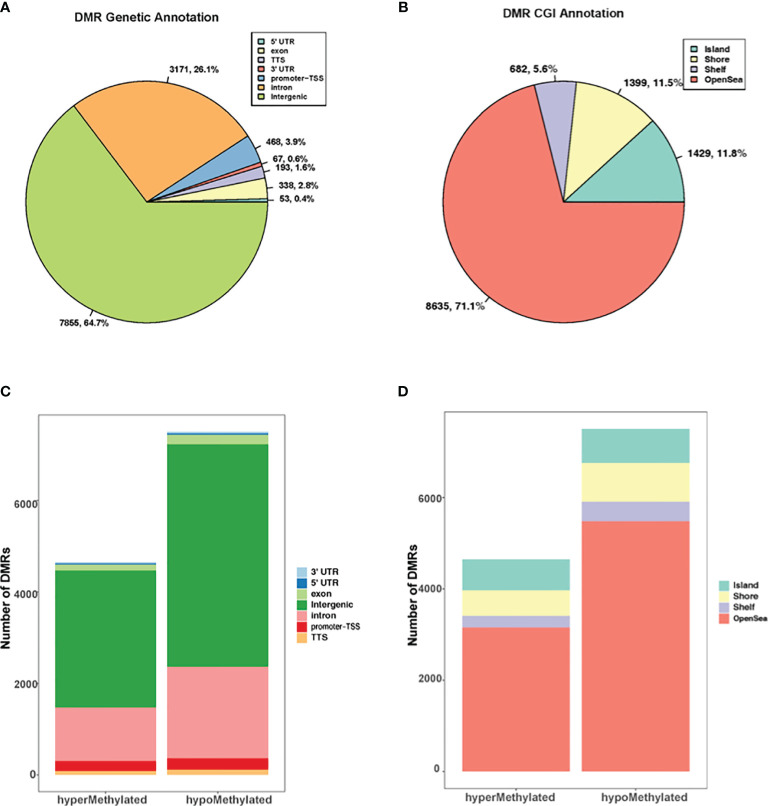
Genome wide distribution of differentially methylated regions (DMRs). **(A)** The proportion of DMRs that overlap with 5’ UTR, exons, TTS, 3’ UTR, promoter-TSS, intron, and intergenic regions. **(B)** The proportion of DMRs with respect to CpG islands versus CpG shores (2000 bp flanking CpG islands), shelves (2000 bp flanking shore), and open sea regions (greater than 4 kb from CpG islands). **(C)** The distribution of hyper and hypomethylated regions with respect to their genetic location to genes. **(D)** The distribution of hyper and hypomethylated regions with respect to their distance to CGIs.

The genomic and CpG island context of the DMRs were investigated in *G. parasuis* infected porcine brain and results showed that the differentially methylated regions were distributed throughout the whole genome (**Figure 1**). DMRs were most common in intergenic regions (64.7%), and less common in introns (26.1%). Only a minor percentage of DMRs occurred in promoters (3.9%), followed by exons (2.8%), transcriptional termination sites (TTS, 1.6%), 3’ untranslated regions (3’ UTR, 0.6%) and 5’ untranslated region (5’ UTR, 0.4%) (**Figure 1A**). The genomic annotation profile exhibited similarities when we focused on hyper- and hypo- methylated DMRs separately. The majority of DMRs were annotated in intergenic regions (64.4% and 64.9% for hypermethylated and hypomethylated DMRs, respectively), and a minor percentage of DMRs were annotated in promoters (4.7% and 3.3% respectively) (**Figure 1C**). Furthermore, the CGI (CpG island) annotation showed that most DMRs were found outside CGIs and most of these were in the open sea regions (greater than 4 kb from CpG islands) (**Figure 1B**). For hypermethylated and hypomethylated DMRs, 14.7% and 10.0% were annotated in CGIs, 12% and 11.2% were in shores (2000 bp flanking CpG islands), and 5.4% and 5.7% were in shelves (2000 bp flanking shore regions) (**Figure 1D**).

### Genome-Wide Transcriptome Analysis

**Figure 2:**
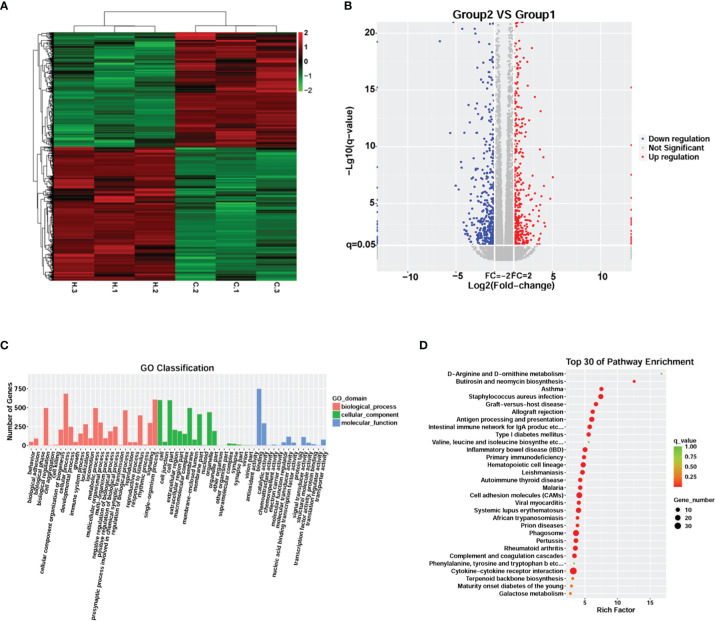
Transcriptome analysis comparing *G. parasuis*-infected porcine brain vs. control porcine brain. **(A)** Hierarchical clustering heatmap of transcript abundance from RNA-seq. Each row represents a transcript, H.1, H.2, H.3 represent *G. parasuis*-infected porcine brain, and C.1, C.2, C.3 represent negative controls. **(B)** Volcano plot showing differentially expressed genes. The vertical axis shows the -log10 (q-value), and the horizontal axis shows log2 (Fold-change). **(C)** GO functional category analysis of differentially expressed genes. The vertical axis shows the number of genes in each term, and the horizontal axis shows biological process, cellular component, and molecular function terms. **(D)** KEGG pathway enrichment of differentially expressed genes. Top 30 KEGG pathways are shown in the vertical axis and the horizontal axis shows the rich factor.

RNA-seq analysis was performed to explore changes in the global transcriptome occurring in meningitis induced by *G. parasuis* infection. Unsupervised hierarchical clustering identified two unique clusters that had distinct signatures (**Figure 2A**). A total of 1,126 genes were significantly differentially expressed in *G. parasuis* infected porcine brain, including 606 up-regulated and 520 down-regulated genes (fold change > 2, adjusted *p* < 0.05) (**Figure 2B** and **Supplementary File 6**).

GO and KEGG enrichment were performed to reveal the potential function of the affected genes involved in the pathogenesis of meningitis induced by *G. parasuis*. GO enrichment showed that cellular processes, single-organism processes, and metabolic processes were the most abundant terms in the biological process category. In the cellular component category, cell, cell part and membrane were the top three terms, while binding, catalytic activity, and molecular transducer activity were the top terms in the molecular function category (**Figure 2C**). KEGG analysis revealed that cytokine-cytokine receptor interaction, cell adhesion molecules (CAMs), and phagosome were the most significantly abundant pathways involved in the pathogenesis of *G. parasuis* induced meningitis (**Figure 2D**).

### Integrated Analysis of DNA Methylation and mRNA Expression

**Figure 3:**
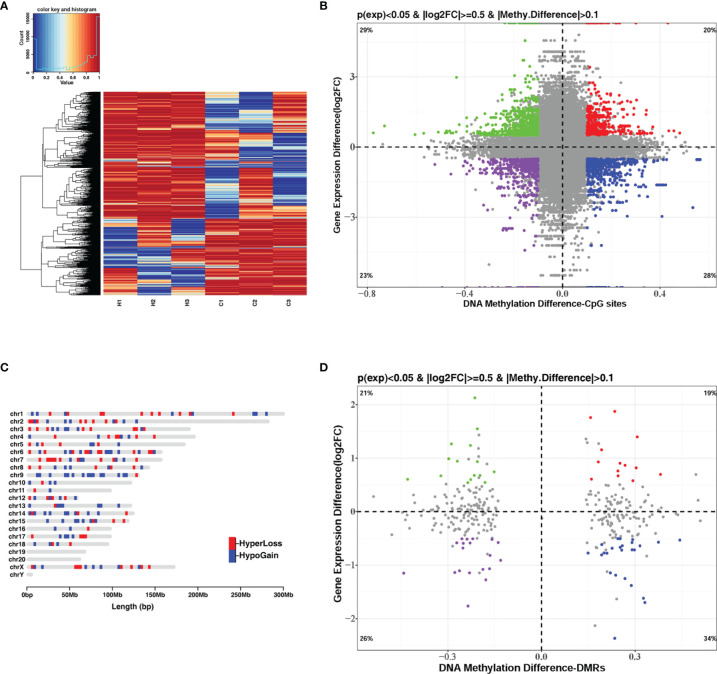
Integrated analysis of DNA methylation and mRNA expression when comparing *G. parasuis*-infected porcine brain vs. control porcine brain. **(A)** Hierarchical clustering heatmap of the top-1000 differentially methylated CpG sites from whole-genome bisulfite sequencing. Each row represents a methylated CpG site, H1, H2, H3 represent *G. parasuis*-infected porcine brain, and C1, C2, C3 represent negative controls. **(B)** Scatter plot integrating CpGs methylation and mRNA expression. The vertical axis shows differences in gene expression and the horizontal axis shows differences in DNA methylation for the CpG sites. 1, 3-quadrants represent positive correlated genes, in which gene up-regulation correlates with hypermethylation and gene down-regulation correlates with hypomethylation. The 2, 4-quadrants represent inversely correlated genes, in which gene up-regulation correlates with hypomethylation and gene down-regulation correlates with hypermethylation. **(C)** Karyogram showing genome-wide coverage of differentially methylated sites that inversely correlate to gene expression (2, 4-quadrants in B). Hypermethylated sites correlated with gene down-regulation are shown in red (HyperLoss). Hypomethylated sites correlated with gene up-regulation are shown in blue (HypoGain). **(D)** Scatter plot integrating DMRs and mRNA expression. The vertical axis shows differences in gene expression and the horizontal axis shows differences in DNA methylation of DMRs. The 1, 3-quadrants represent positively correlated genes and the 2, 4-quadrants represent inversely correlated genes.

**Figure 4:**
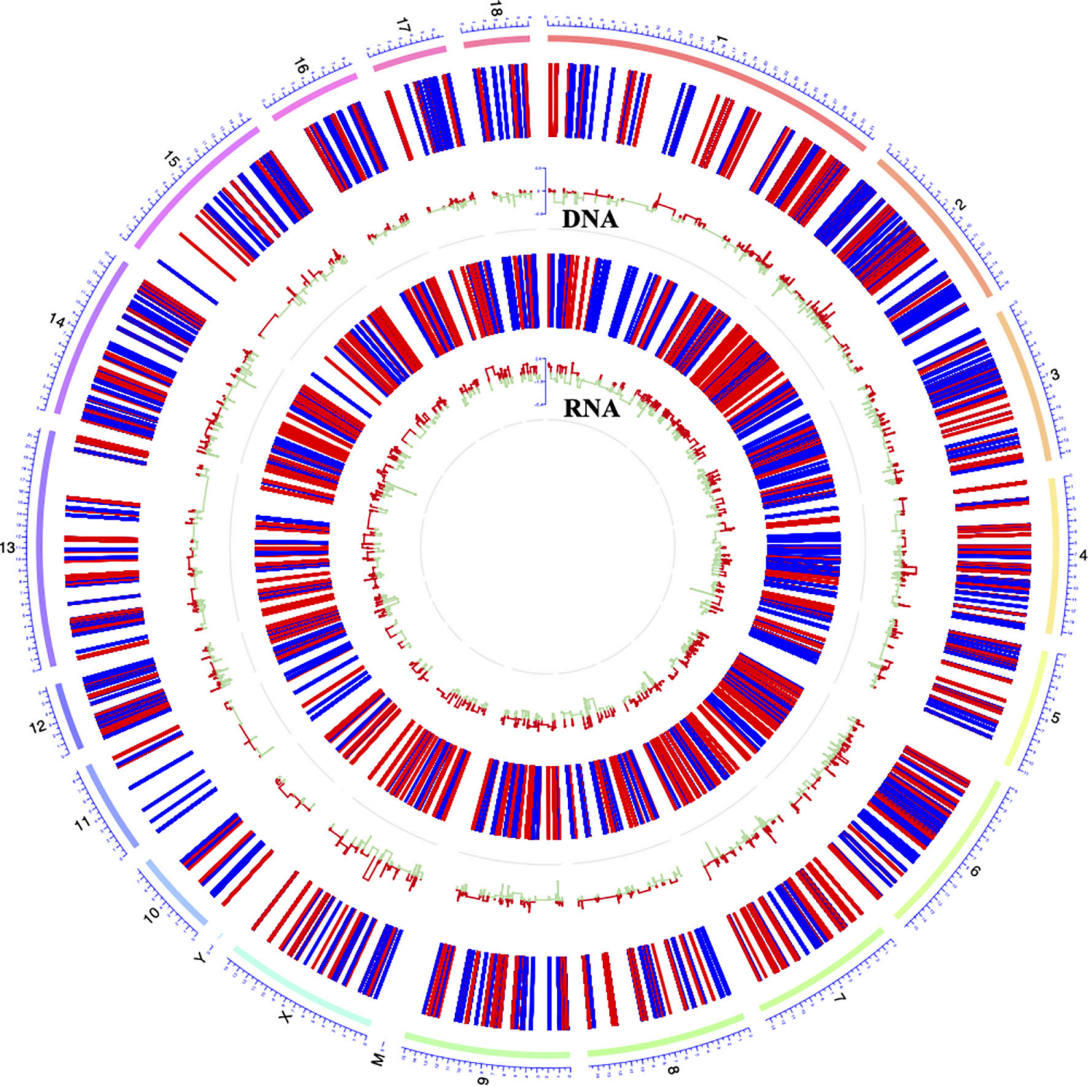
Circos plot showing DNA methylation and mRNA transcription on pig chromosomes. The outermost circle displays the pig chromosomes; The second layer from the outside represents the genome-wide distribution of differentially methylated CpGs in promoter regions; The innermost layer represents the differentially expressed mRNAs identified by RNA-seq. Red bars indicate hypermethylation or up-expression, and blue/green bars indicate hypomethylation or down-expression.

DNA methylation and gene transcription are normally causal related, where hypermethylation might be associated with gene down-regulation and hypomethylation might be related to gene up-regulation, especially when methylated sites are located in the promoter region of genes. The hierarchical clustering of the top-10000 significantly differentially methylation sites, considering different genomic context (5’UTR, 3’UTR, exon, intergenic, intron, promoter-TSS, and TSS), showed a striking difference between the control and *G. parasuis* infected samples (**Figure 3A** and **Supplementary Figure 1**). A volcano plot showed the distribution of different methylation sites under different methylation thresholds (**Supplementary Figure 2**). To further identify the significance of DNA methylation in the regulation of gene expression, we integrated the CpGs methylation patterns with the gene expression profiles obtained from the same sample. The differentially methylated CpG sites in the promoter region and the differentially expressed mRNA transcripts were aligned to chromosomes throughout the whole genome (**Figure 4** **)**. The integrated analysis showed that 301 differentially expressed genes corresponding to 1068 differentially methylated CpGs in their promoter regions exhibited an inverse correlation (|log_2_FC| > 0.5, |diffMethy| > 0.1, and *P* < 0.05) (**Figure 3B** and **Supplementary File 7**). Furthermore, 141 genes (28%) were significantly hypermethylated and down-regulated, while 160 genes (29%) were significantly hypomethylated and up-regulated (|log_2_FC| > 0.5, |diffMethy| > 0.1, and *P* < 0.05) (**Figure 3B**). The inversely correlated methylation sites were detected throughout the genome on all chromosomes except chromosome 19, 20 and chromosome Y (**Figure 3C**).

The same integrated analysis was performed on the DMRs and gene expression profiles. Forty DEGs corresponding to 40 DMRs in the promoter regions were inversely associated, and 33 DEGs were positively correlated with their promoter DMRs (|log_2_FC| > 0.5, |diffMethy| > 0.1, and *P* < 0.05) (**Figure 3D** and **Supplementary Files 8**, **9**). **Table 1** shows the top 30 significant DEGs with an inverse correlation between DMR and gene expression.

**Table 1:** Top 30 genes with an inverse correlation between DMR and gene expression in *G. parasuis-*infected porcine brain.

Gene name	Gene ID	Log_2_FC	Diff.Methy	*P*-value	Location	nCpG	Length (bp)
SEMA4D	ENSSSCG00000009584	1.2656034	-0.2883937	2.03E-91	promoter	15	256
KCNA1	ENSSSCG00000000716	1.23644958	-0.2273311	2.78E-59	promoter	5	55
ARHGAP23	ENSSSCG00000018058	0.94406544	-0.2694318	8.76E-53	promoter	13	206
GBP1	ENSSSCG00000006924	-1.1858318	0.23790912	7.35E-52	promoter	6	103
FRMD1	ENSSSCG00000004017	2.12614016	-0.213235	1.36E-34	promoter	12	107
VWA1	ENSSSCG00000003344	1.54657552	-0.2053669	1.58E-26	promoter	14	236
SYP	ENSSSCG00000012292	-0.6355327	0.25905489	3.45E-26	promoter	17	291
EPHX2	ENSSSCG00000009666	0.60089938	-0.2265636	1.67E-21	promoter	28	802
NGEF	ENSSSCG00000016295	-0.6819392	0.22641751	1.06E-20	promoter	8	183
CCDC136	ENSSSCG00000016579	-0.7114116	0.29046062	1.22E-20	promoter	6	127
MDH2	ENSSSCG00000025486	-0.6379805	0.31565165	1.28E-16	promoter	7	203
TET3	ENSSSCG00000008292	0.60620955	-0.4286807	4.98E-16	promoter	18	110
EFCAB14	ENSSSCG00000003895	0.54650846	-0.180695	2.66E-14	promoter	25	418
SFT2D1	ENSSSCG00000004020	0.67734274	-0.1966982	5.48E-14	promoter	12	62
ACVR1B	ENSSSCG00000000233	-0.5302004	0.4433712	6.39E-12	promoter	20	103
C1QA	ENSSSCG00000003524	-1.144042	0.22095373	7.08E-11	promoter	4	233
DTNBP1	ENSSSCG00000001062	-0.7755109	0.20987304	1.13E-09	promoter	26	287
PITPNM1	ENSSSCG00000012903	-0.5045044	0.25220708	1.75E-09	promoter	43	104
SGCE	ENSSSCG00000015328	0.74597466	-0.1510948	4.08E-09	promoter	23	91
ADA	ENSSSCG00000021255	0.98787544	-0.2969333	3.62E-08	promoter	8	372
STXBP2	ENSSSCG00000013576	-1.6173215	0.3266804	1.93E-07	promoter	20	316
HS6ST2	ENSSSCG00000021375	-0.5033069	0.24492105	8.92E-07	promoter	11	76
CH242-402I11.1	ENSSSCG00000003395	-0.7706879	0.20045268	1.64E-06	promoter	4	85
SFI1	ENSSSCG00000010031	-0.7724897	0.14949271	2.08E-06	promoter	18	114
SLC35B2	ENSSSCG00000001702	0.54376229	-0.2357549	3.87E-06	promoter	5	88
LYPD1	ENSSSCG00000015706	-0.6140079	0.19323329	7.19E-06	promoter	30	155
TPM2	ENSSSCG00000005316	-0.6653554	0.25232273	1.97E-05	promoter	6	86
ARX	ENSSSCG00000020801	-0.7139706	0.29808345	3.98E-05	promoter	6	73
SLC31A2	ENSSSCG00000023714	0.93577312	-0.2053315	0.00012846	promoter	6	124
FADS3	ENSSSCG00000013073	-0.5689346	0.37487823	0.00031746	promoter	21	163

Log_2_FC: Log_2_(Fold Change).

Diff.Methy, difference of methylation.

nCpG: number of CpG site.

P-value, the significance for the gene expression difference.

In addition to the inversely correlated genes, we also observed an equivalent number of positively correlated genes, which were hypermethylated but up-regulated or hypomethylated but down-regulated (**Figures 3B, D** **)**. Thus, both negative and positive correlations between DNA methylation and mRNA expression were observed in our *G. parasuis* infected porcine brain samples, suggesting that there may be two different mechanisms involving DNA methylation-dependent gene regulation for *G. parasuis* induced meningitis.

### Identification of DNA Methylation-Related Biological Pathways

**Figure 5:**
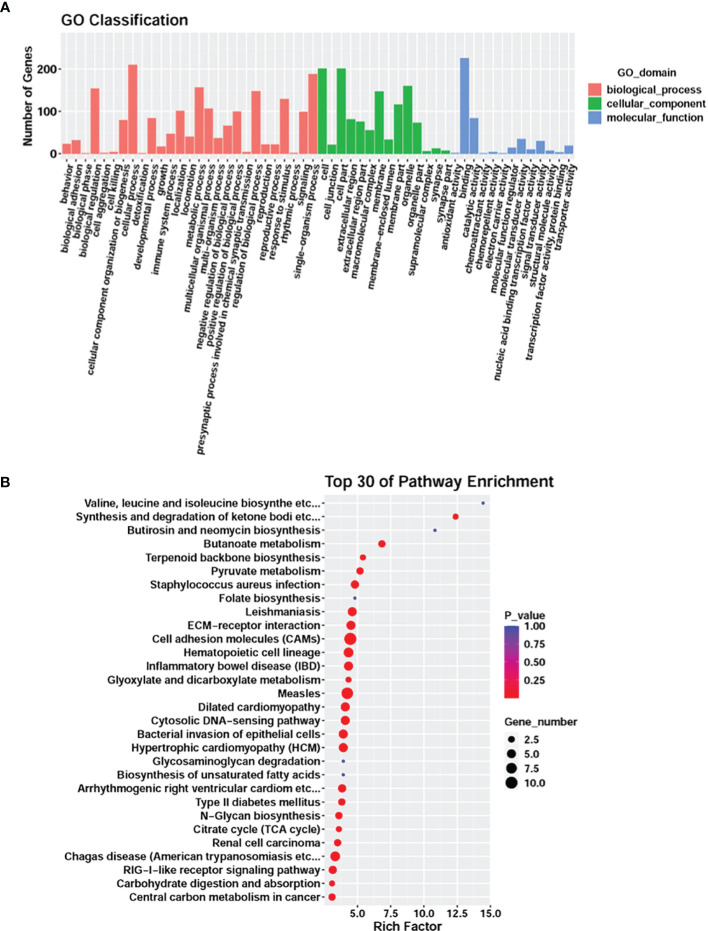
GO functional category analysis and KEGG pathway enrichment of the inversely correlated genes in *G. parasuis*-infected porcine brain. **(A)** GO functional category analysis of inversely correlated genes. The vertical axis shows the number of genes in each term, and the horizontal axis shows biological processes, cellular component, and molecular function terms. **(B)** KEGG pathway enrichment of inversely correlated genes. Top 30 KEGG pathways are shown in the vertical axis and the horizontal axis shows the rich factor.

GO and KEGG enrichment were performed to reveal the biological dysfunctions caused by the DNA methylation and the expressed inversely correlated genes in *G. parasuis* infected samples. GO enrichment showed that cellular processes, single-organism processes, and metabolic processes were the most abundant terms in the biological process category. In the cellular component category, cell, cell part, and organelle were the top three terms, while binding, and catalytic activity were the top terms in the molecular function category (**Figure 5A**). When compared to the KEGG pathways enriched by all the differential genes (**Figure 2D**), cell adhesion CAMs, and hematopoietic cell lineages were common between the two enrichments, and bacterial invasion of epithelial cells, and RIG-1-like receptor signaling pathways were particularly enriched by the inversely correlated genes in *G. parasuis* infected porcine brain (**Figure 5B**).

### Validation of DNA Methylation and Gene Expression

**Figure 6:**
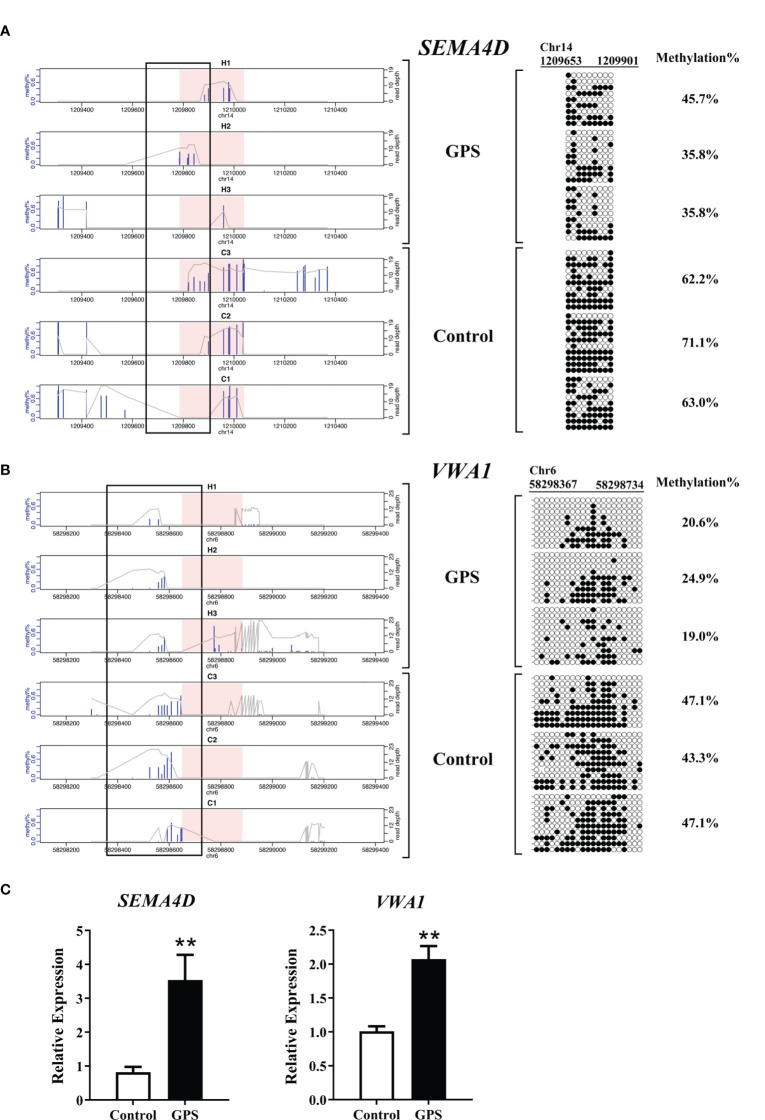
Bisulfite sequencing and qRT-PCR validation of *SEMA4D* and *VWA1* in *G. parasuis*-infected vs. control porcine brain. **(A)** Bisulfite sequencing validation of *SEMA4D.* On the left, bisulfite sequencing depth shows the *SEMA4D* DMR and adjacent flaking regions in three *G. parasuis*-infected and three negative control samples. On the right, targeted bisulfite sequencing validation of a region overlapping the *SEMA4D* DMR as indicated by the rectangle over the sequencing tracks. Each row represents a single clone. Dark circles represent methylated and open circles represent unmethylated cytosines. **(B)** Bisulfite sequencing validation of *VWA1.* Similarly, bisulfite sequencing depth for *VWA1* DMR and adjacent flaking regions is shown on the left, and bisulfite sequencing validation is shown on the right. **(C)** qRT-PCR validation of *SEMA4D* and *VWA1*. The vertical axis indicates the relative expression levels of *SEMA4D* or *VWA1* in reference to β-actin, and the horizontal axis indicates the *G. parasuis*-infected vs. negative control samples. **P < 0.01.

To determine the reliability of the sequencing data, mRNA expression and DNA methylation at base-pair resolutions were examined on two inversely correlated genes, semaphoring 4D (*SEMA4D*) and von Willebrand factor A domain containing 1 (*VWA1*), according to the significance criteria. Bisulfite sequencing showed that the methylation rate in the DMR of *SEMA4D* decreased from 65.4% to 39.1% after *G. parasuis* stimulation, while the methylation rate in the DMR of *VWA1* decreased from 45.8% to 21.5% after *G. parasuis* stimulation (**Figures 6A, B** **)**. Validation with RT-PCR showed that the mRNA expression of *SEMA4D* and *VWA1* were significantly up-regulated after *G. parasuis* stimulation (*P* < 0.01), consistent with the hypomethylation in the promoter regions of each gene (**Figure 6C**).

### PPI Network of Differential Genes

**Figure 7:**
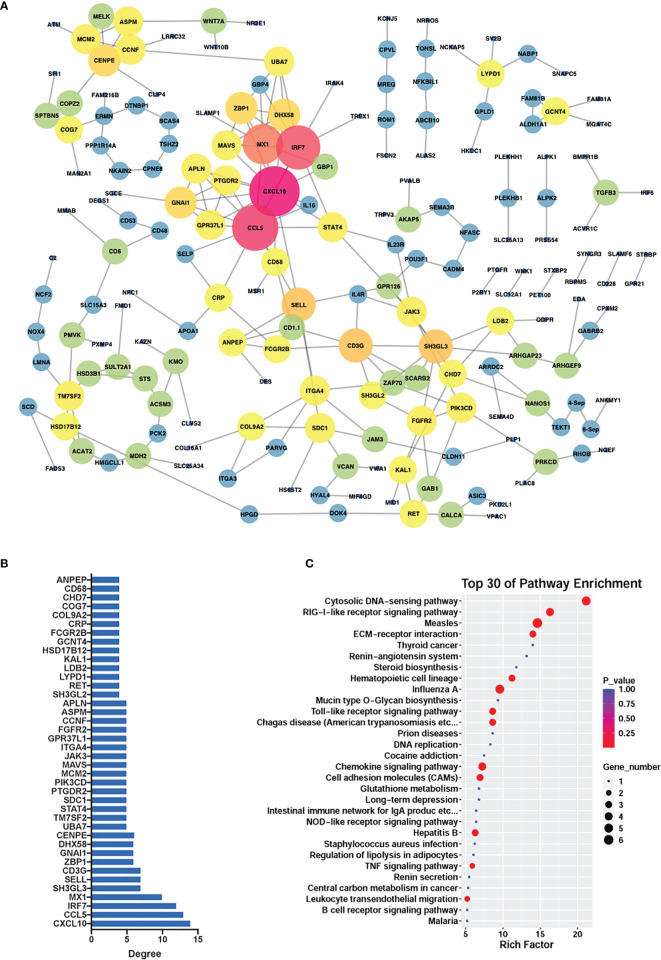
PPI network. **(A)** PPI network constructed from significantly differentially expressed and methylated genes in *G. parasuis*-infected porcine brain. Nodes represent genes and edges represent interactions. The node size represents the node degree. **(B)** The top 40 hub genes with the highest node degrees corresponding to the network. The vertical axis shows gene name, and the horizontal axis shows the node degree. **(C)** KEGG pathway enrichment of the top 40 hub genes. Top 30 KEGG pathways are shown in the vertical axis and the horizontal axis shows the rich factor.

To reveal the potential associations within the differentially methylated genes, we created a protein-protein interaction network which consisted of 193 protein nodes and 243 interactions based on the differential genes (|log_2_FC| > 0.5, |diffMethy| > 0.1, and *P* < 0.05) (**Figure 7A**), with *CCL5* and *CXCL10* as the core genes. The crucial roles of genes were determined by the node degree and the top 40 genes with the highest connection degrees were selected and subsequently used for KEGG pathway enrichment (**Figure 7B**). The KEGG result showed that the top 40 genes mainly participated in chemokine signaling pathways, cytosolic DNA-sensing pathways, RIG-1-like receptor signaling pathway, Toll-like receptor signaling pathway, CAMs signaling pathways, and TNF signaling pathway (**Figure 7C**).

## Discussion

Genome-wide DNA methylation has been shown to be associated with a variety of diseases, and the host DNA methylation profile can be altered upon exposure of cells to environmental pathogens (14, 18). Intensive studies have been performed looking at the pathogenic mechanism of *G. parasuis* infection, but the genome-wide DNA methylation pattern as well as the relationship between DNA methylation and gene expression during the process of infection is still unknown. To our knowledge, this study represents the first attempt to profile a genome-wide DNA methylation pattern and integrate both DNA methylome and transcriptome data from porcine brain to identify key genes and pathways that are possibly regulated by DNA methylation during the process of *G. parasuis* infection. This work may provide information for the development of epigenetic biomarkers and potential therapeutic target for *G. parasuis* induced meningitis.

Genomic location of DNA methylation has a major effect to the type of function produced by this epigenetic modification (12, 14). Thus, we investigated the genomic and CpG island contexts of the DMRs in *G. parasuis* infected porcine brain. Our results found that DMRs are most frequent in intergenic regions and introns, with only a comparatively low percentage of DMRs occurring in promoters and CpG islands, which is consistent with other report of the distribution patterns of DNA methylation in other diseases (29, 30). Intergenic regions contain cis factors that participate in the regulation of gene transcription during pathogen infection and inflammatory responses (12, 14). Published data indicate that DNA methylation within introns may participate in alternative exon splicing (31). Whereas DNA methylation outside of CGIs in shores/shelves may regulate expression by creating alternative transcriptional start sites (32). Research on human dendritic cells reported that bacterial infection-induced demethylation rarely occurs at promoter regions and instead localizes to distal enhancer elements, some of which regulate the activation of immune transcription factors (33). Therefore, DMRs in distal regulatory elements may also have meaningful functions in gene regulation. The large amount and genome-wide distribution of differential DNA methylation observed in our data indicate that this epigenetic modification may play an important role during the process of *G. parasuis* infection.

Whether *de novo* DNA methylation is the reason for gene silencing or a result of gene regulation is still debated but increasing evidence has indicated that there are causal associations between DNA methylation changes and phenotypes (34, 35). It has been reported that methylated CpGs can prevent the binding of some transcription factors (12), and DNA methylation can induce a more compact and rigid nucleosome structure and regulate alternative polyadenylation of mRNA during transcription (36, 37). Therefore, it is well accepted that DNA methylation and gene transcription is inversely correlated, with hypermethylation associated with gene down regulation and that hypomethylation is associated with gene up regulation. However, in our integrated analysis, the amount of inversely correlated genes and positively correlated genes were similar, suggesting that the relationship between DNA methylation and gene transcription may be more complicated than our previous expectation. The regulatory mechanism involved in DNA methylation and gene expression may vary depending on the genomic location of the methylated sequence or site (12, 14).

To explore the potential functions of these differentially expressed genes and provide new perspectives on the function of DNA methylation, we performed KEGG pathway enrichment analysis on all DEGs and DNA methylation-mRNA expression inversely correlated genes. After comparing the results, we found CAMs, and hematopoietic cell lineages were common between the two enrichments, with cytokine-cytokine receptor interaction specifically appearing in the pathways enriched by all DEGs and bacterial invasion of epithelial cells, and RIG-1-like receptor signaling pathways specifically identified in the results from inversely correlated genes. During the pathology of bacterial meningitis, which is an inflammatory disease of the CNS, bacterial epithelial cell adhesion and invasion is an important step followed by invasion and break down of the blood-brain barrier (BBB) (38, 39). The first step in the interaction of blood-born bacteria with the brain endothelium is adherence/attachment to brain microvascular endothelial cells, in which CAMs and receptors bind to multiple ligands from the bacterial surface and act as a bridges (40). Thus, we infer those changes in DNA methylation that are inversely associated with the regulation of gene expression may participate in the process of bacterial invasion of epithelial cells during *G. parasuis* infection and finally result in meningitis. Further experiments however are needed in porcine brain microvascular endothelial cells.

We also constructed a protein-protein interaction network among differentially methylated and expressed genes (|log_2_FC| > 0.5, |diffMethy| > 0.1, and *P* < 0.05), in which *CCL5* and *CXCL10* were identified as the core genes of the network. *CCL5* (chemokine ligand 5) and *CXCL10* (CXC motif chemokine ligand 10) are chemokines responsible for facilitating inflammatory responses, including the adhesion and migration of different T cell subsets in immune responses, and are involved in various pathological processes including inflammation, chronic diseases, and cancers as well as the infection of COVID-19 (41, 42). Interestingly, as key genes in the cytokine-cytokine receptor interaction signaling pathway, *CCL5* and *CXCL10* were also identified as core genes in our previous study in porcine aortic vascular endothelial cells infected with *G. parasuis* (19), and this prompted us to infer that these two chemokines may play a very important role in the immune response during *G. parasuis* infection and further study is necessary to confirm this.

For the inversely correlated genes, we validated *SEMA4D* and *VWA1*, for which hypomethylation in the promoter region of both genes was associated with gene up regulation in *G. parasuis* infected porcine brain. *SEMA4D* is a classic member of the semaphorin family that is widely represented in the immune system and plays an important role in many physiological and pathological processes, including immunoregulation, angiogenesis, neurogenesis, and tumor progression (43, 44). It is reported that *SEMA4D* contributes to the formation of cellular, humoral, and innate immune responses, such as inflammation, immune cell migration, and immunological synapse formation (45). It has been demonstrated that *SEMA4D* plays an important role in angiogenesis by promoting chemotaxis of endothelial cells (46). Although the effect of *SEMA4D* in the pathogenesis of meningitis has not been reported, since the expression of *SEMA4D* is up regulated with hypomethylation in the promoter region after *G. parasuis* infection, we speculate that *SEMA4D* may play an important role in immunoregulation and inflammation during this process. However, further study is needed to validate this hypothesis. *VWA1* encodes the von Willebrand factor A domain containing 1 protein, which is an extracellular matrix protein expressed in muscle and peripheral nerves but whose function is poorly understood (47, 48). Lack of VWA1 is known to compromise peripheral nerve structure and function in a *VWA1* knock-out mouse model (49). Exome sequencing identified bi-allelic loss of function variants in *VWA1* as the molecular basis of neuromyopathy, a neuromuscular disorder (50). Hypermethylation of a non-coding region 1.1 kb upstream from the *VWA1* gene has been reported to be associated with clear-cell ovarian cancer (51). These results demonstrating that hypomethylation in the promoter region of *VWA1* and its gene up-regulation in *G. parasuis* infected porcine brain provides a new understanding of the potential function of *VWA1*.

Our study is the first attempt to integrate DNA methylome and transcriptome data from *G. parasuis* infected porcine brains and has found that *G. parasuis* can induce changes to whole-genome DNA methylation and gene expression profiles in porcine brain. The network and pathways identified by the DNA methylation-gene expression correlated genes suggest that key sites of differential DNA methylation could lead to molecular aberrations underlying meningitis and provide potential targets for further pathological study. Our data also provides a basis for defining the contribution of genome-wide DNA methylation to the pathogenesis of meningitis in pigs and provide information for the development of epigenetic biomarkers and potential therapeutic target for *G. parasuis* induced meningitis.

## Data Availability Statement

The data used in this study was deposited in the NCBI Sequence Read Archive (SRA) repository, accession number PRJNA800576.

## Ethics Statement

The animal study was reviewed and approved by Animal Care and Use Committee of Wuhan Polytechnical University, Hubei Province, China (EM947, 5th November 2020); Hubei Province Science and Technology Department of China (permit number SYXK[ER] 2010-0029).

## Author Contributions

YQ and LG conceived and designed the experiments. LG, HXC, SF, JL, and YZ performed the experiments. LG, HXC, SF, and HBC analyzed the data. LG, SF, and YQ wrote the paper. All authors contributed to the article and approved the submitted version.

## Funding

This work was supported by the National Natural Science Foundation of China (grant no. 32072917), science and technology research program of Hubei Provincial Department of Education (No. D20211606), Key Laboratory of Animal Embryo Engineering and Molecular Breeding of Hubei Province (KLAEMB-2019-03), and key laboratory of prevention and control agents for animal bacteriosis (Ministry of Agriculture and Rural Affairs) (KLPCAAB-2020-01).

## Conflict of Interest

The authors declare that the research was conducted in the absence of any commercial or financial relationships that could be construed as a potential conflict of interest.

## Publisher’s Note

All claims expressed in this article are solely those of the authors and do not necessarily represent those of their affiliated organizations, or those of the publisher, the editors and the reviewers. Any product that may be evaluated in this article, or claim that may be made by its manufacturer, is not guaranteed or endorsed by the publisher.

## Supplementary Material

Supplementary Figure 1 Hierarchical clustering heatmaps of differentially methylated CpG sites considering genomic context. Hierarchical clustering heatmap of differentially methylated CpG sites in 3’UTR **(A)**, 5’UTR **(B)**, exon **(C)**, intergenic **(D)**, intron **(E)**, promoter-TSS **(F)**, TTS **(G)**. Each row represents a methylated CpG site, H1, H2, H3 represent *G. parasuis*-infected porcine brain, and C1, C2, C3 represent negative controls.

Supplementary Figure 2 Volcano plot showing differentially methylated CpGs at different DNA methylation difference thresholds. The vertical axis shows the -log10 (p-value), and the horizontal axis shows DNA methylation difference. Blue dots shows the hypo-methylated CpGs when DNA methylation difference threshold is 0.1, red dots shows the hyper-methylated CpGs when DNA methylation difference threshold is 0.1
