# Supplementary figures and images for: Methylome and Transcriptome-Based Integration Analysis Identified Molecular Signatures Associated With Meningitis Induced by Glaesserella parasuis
Source: Front Immunol. 2022 Feb 25;13:840399. doi: 10.3389/fimmu.2022.840399 (PMC8913945; doi:10.3389/fimmu.2022.840399)

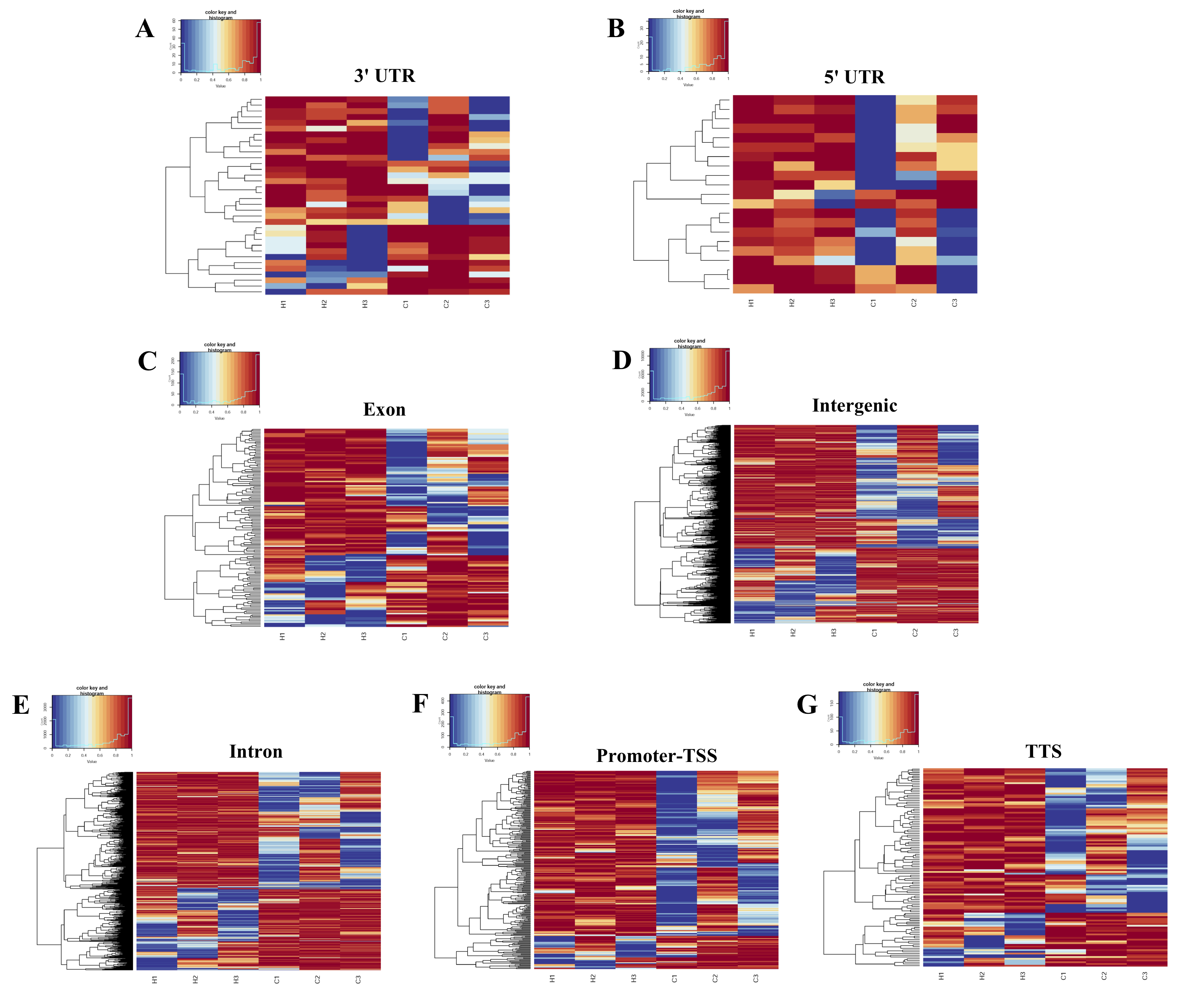

Supplement: Supplementary Figure 1 — Hierarchical clustering heatmaps of differentially methylated CpG sites considering genomic context. Hierarchical clustering heatmap of differentially methylated CpG sites in 3’UTR (A), 5’UTR (B), exon (C), intergenic (D), intron (E), promoter-TSS (F), TTS (G). Each row represents a methylated CpG site, H1, H2, H3 represent G. parasuis-infected porcine brain, and C1, C2, C3 represent negative controls. [file Image_1.tiff]

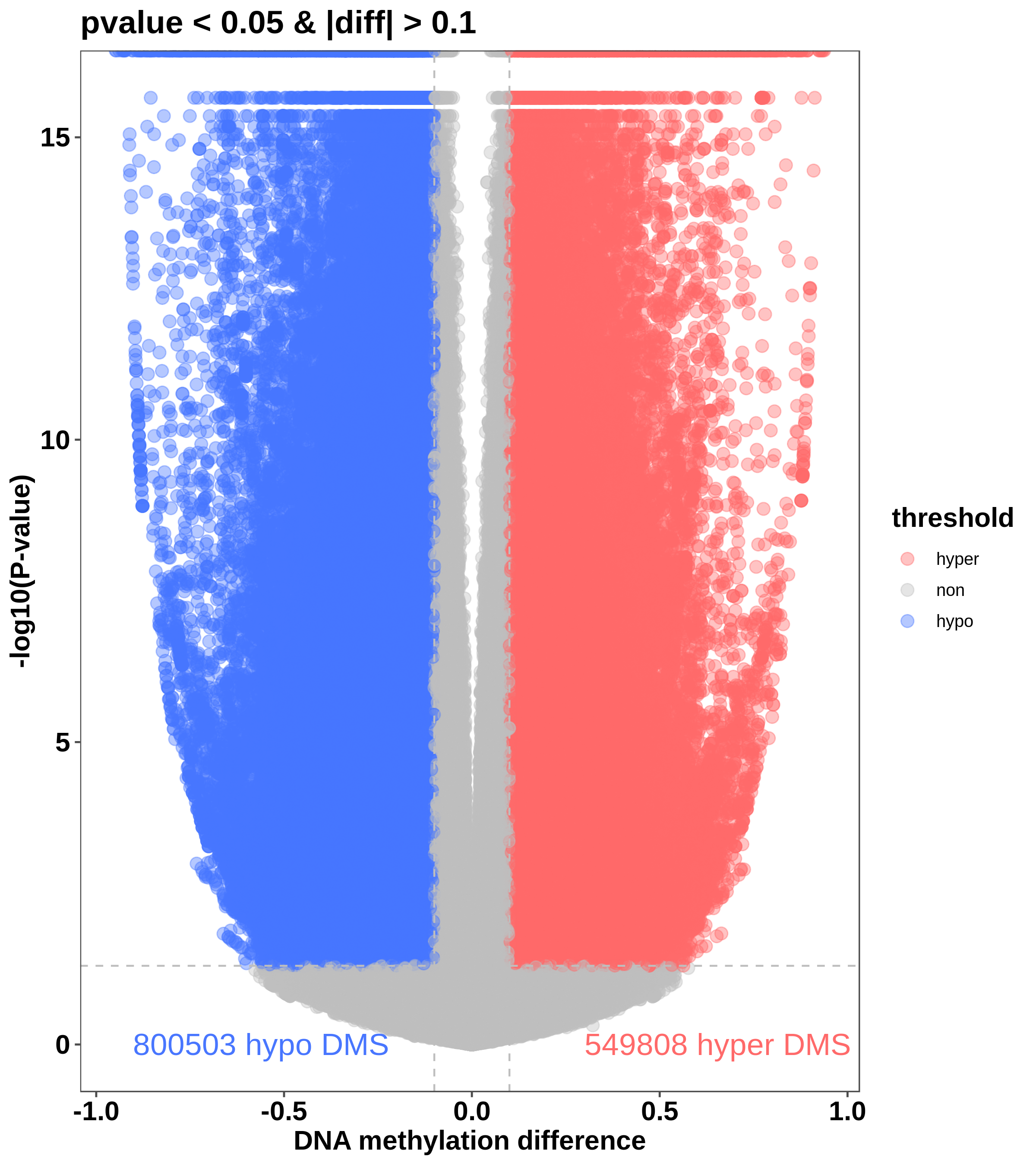

Supplement: Supplementary Figure 2 — Volcano plot showing differentially methylated CpGs at different DNA methylation difference thresholds. The vertical axis shows the -log10 (p-value), and the horizontal axis shows DNA methylation difference. Blue dots shows the hypo-methylated CpGs when DNA methylation difference threshold is 0.1, red dots shows the hyper-methylated CpGs when DNA methylation difference threshold is 0.1 [file Image_2.tiff]
