# Supplementary material for: Methylome and Transcriptome-Based Integration Analysis Identified Molecular Signatures Associated With Meningitis Induced by Glaesserella parasuis
Source: Front Immunol. 2022 Feb 25;13:840399. doi: 10.3389/fimmu.2022.840399 (PMC8913945; doi:10.3389/fimmu.2022.840399)
Supplement: Supplementary file 3 [file DataSheet_1.docx]

**Supplementary file 1. Primers for Bisulfite Sequencing.**

| Gene | Nucleotide Sequence (5'-3') | | Tm (℃) | Length (bp) |
| --- | --- | --- | --- | --- |
|  |  |  |  |  |
| SEMA4D | Forward | GGGGTAAATTTTAGTGTTTAATTGT | 59 | 762 |
|  | Reverse | CTACTCTCACCACATCCCCAA |  |  |
| VWA1 | Forward | GTTTAGTTATAATTTTAGATGTAAGGTTTT | 59 | 433 |
|  | Reverse | CCCACTACCCTTATCTCTACAATAC |  |  |
