# Supplementary material for: Methylome and Transcriptome-Based Integration Analysis Identified Molecular Signatures Associated With Meningitis Induced by Glaesserella parasuis
Source: Front Immunol. 2022 Feb 25;13:840399. doi: 10.3389/fimmu.2022.840399 (PMC8913945; doi:10.3389/fimmu.2022.840399)
Supplement: Supplementary file 4 [file DataSheet_2.docx]

**Supplementary file 2. Primers for quantitative RT-PCR.**

| Gene | Nucleotide Sequence (5'-3') | | Tm (℃) | Length (bp) |
| --- | --- | --- | --- | --- |
|  |  |  |  |  |
| SEMA4D | Forward | CCAACAGACGGCTCACAGACCA | 60 | 280 |
|  | Reverse | CAGAACGCATCCTCACGCACAC |  |  |
| VWA1 | Forward | GCCACTCAGGTCCTGGATGTTG | 60 | 283 |
|  | Reverse | GCCTTCACGCCTTCCGATCT |  |  |
| β-actin | Forward | TGCGGGACATCAAGGAGAAG | 58 | 216 |
|  | Reverse | AGTTGAAGGTGGTCTCGTGG |  |  |
