# Supplementary material for: Methylome and Transcriptome-Based Integration Analysis Identified Molecular Signatures Associated With Meningitis Induced by Glaesserella parasuis
Source: Front Immunol. 2022 Feb 25;13:840399. doi: 10.3389/fimmu.2022.840399 (PMC8913945; doi:10.3389/fimmu.2022.840399)
Supplement: Supplementary file 5 [file DataSheet_3.doc]

**Supplementary file 3. The statistics of whole-genome bisulfite sequencing in porcine brain**

| **Sample ID** | **Raw reads** | **Clean reads** | **Clean ratio (%)** | **Uniquely Mapped reads** | **Uniquely Mapping ratio (%)** | **Conversion ratio (%)** |  |
| --- | --- | --- | --- | --- | --- | --- | --- |
| Control 1 | 596,457,794 | 584,615,613 | 98.2 | 317,170,658 | 54.3 | 99.3 |  |
| Control 2 | 668,068,227 | 659,907,074 | 97.6 | 393,901,578 | 59.7 | 99.1 |  |
| Control 3 | 618,743,245 | 608,501,462 | 96.1 | 344,934,382 | 56.7 | 99.3 |  |
| HPS 1 | 597,732,790 | 584,255,710 | 99.0 | 318,373,406 | 54.5 | 99.4 |  |
| HPS 2 | 681,215,521 | 672,269,078 | 98.7 | 407,902,980 | 60.7 | 99.4 |  |
| HPS 3 | 600,563,799 | 586,894,730 | 99.0 | 328,116,854 | 55.9 | 99.3 |  |
| Group 1 (Control 1, 2, 3): normal porcine brain; Group 2 (HPS 1, 2, 3): *G. parasuis*-infected porcine brain. | | | | | | | |
